# T. L. Brown, M. D.—in Memoriam

**Published:** 1887-10

**Authors:** 


### T. L. BROWN, M. D.—IN MEMORIAM.

Dr. Titus L. Brown died of apoplexy at his home in Binghamton, N. Y., August 17th.

Dr. Brown was a physician of considerable ability, of a warm, generous disposition, and will leave many friends to mourn his sudden death.

He was a member of various medical societies, among them of the Central New York Homœopathic Society and the I. H. A.

